# Research on the Method of Digital Media Content Creation Based on the Internet of Things

**DOI:** 10.1155/2022/8529875

**Published:** 2022-04-26

**Authors:** Yiming Sun

**Affiliations:** Fine Arts Academy, Changchun Normal University, Changchun, Jilin 130031, China

## Abstract

This paper provides an in-depth study and analysis of the approach to digital media content creation using IoT technology. This study first investigates both WSN and NB-IoT technologies separately and then combines them to investigate the networking structure based on NB-IoT and WSN as well as the related technologies based on fusion. Then, the coverage method of the node redeployment of the traditional network of wireless sensors is elaborated. For the traditional method, a series of problems such as poor node connectivity and low coverage caused by not conducting a local search of subgroup nodes are proposed, and the node redeployment method of wireless sensor network based on the frog hopping algorithm is proposed, introducing the frog hopping algorithm, combining local search of subgroup and global information exchange, and proposing that for wireless sensor network nodes. We propose initializing the nodes of the wireless sensor network, combining the global information exchange with the subgroup, determining the range of the detectable area of the wireless sensor, and solving the calculus method for the irregular area to get the optimal network nodes so that the nodes of the wireless sensor network can be redeployed. In terms of system, this design abandons the CIS structure, which is more used in the current IoT and uses B/S structure and Java-web technology to design a web-based system. SSH (Struts + Spring + Hibernate) framework, AJAX asynchronous request technology, and JXLL/JFreechart technology are fully applied to improve the practicality and comfort of the system, reduce development and maintenance costs, and facilitate popularization. After the system design was completed, the modules were tested with the existing experimental conditions. The experimental results show that the system is designed to be reliable and can operate normally with high stability and practical significance.

## 1. Introduction

The Internet of Things (IoT) is an extension and expansion of the Internet and communication networks, which mainly identifies the physical world through intelligent devices and sensing technologies, and carries out knowledge mining, processing, and computing based on network transmission and interconnection, to achieve the goal of seamless linkage and information exchange between things and things, and things and people, and thus accurately manage and control the physical world in real time based on scientific decision-making [1]. Since then, the Internet of Things (IoT) has received increasing attention from countries around the world, and the current consensus is that IoT belongs to another round of information technology achievements after mobile communication networks, the Internet, and computer, which is extremely disruptive and revolutionary [2]. Former US President and the first black president Barack Obama responded to the “Smart Planet” strategy (proposed by IBM) as early as 2009, and during his term of office, the IoT strategy was fully integrated into the US national development strategy. The EU IoT Action Plan is the world's first IoT development strategy, which was officially released by the EU in 2009 and describes the trends in IoT technology development and application as its basic content [3]. Advances in science and technology have created new platforms and spaces for artists, greatly enriching the forms of artistic creation and promoting the development of art [4]. The rapid development of technology and media has led to a surprising collision between digital media technology and art in the era of mobile intelligence, and art creation has since had more new forms of expression and development platforms, while the reading needs of the general audience have given art unlimited creative impetus and inspiration [4]. It is widely used in various exhibition halls, digital display, 3D virtual display, interactive display, guide system design, and other aspects of the use of digital media art. In recent years, digital media art has progressed particularly fast in display design activities, and the use of digital TV, digital broadcasting, digital advertising, virtual reality technology, laser light, heat-sensing equipment, etc. has become more widespread, which plays a vital role in improving the visual effect of the display, creating atmosphere, and promoting publicity [5].

The significance of this paper is to use the performance and planning of IoT-NB coverage as a basis to lay a solid foundation for future studies on coverage in other frequency bands, and to serve as a reserve for both coverage enhancement and interference prevention in the future. IoT-NB can be considered as a simplified LTE network, which is characterized by wide coverage area and large connections, and it plays a key role in the analysis and evaluation of IoT-NB IoT coverage and the planning of network capacity and scale [6]. The simulation and calibration of the model in this paper can be used as a guide for the subsequent planning, which is very important in the process of building the network. Today, global digitization is becoming more evident, and under this premise, the need for mobile communications is relatively high, and the efficiency and capability of communications are being enhanced [7]. 5G is a technology characterized by high reliability, low latency, and low power loss, which is no longer possible with simple wireless access technologies, which means that most of the new wireless access technologies need to be integrated to provide a combined solution. Combining new wireless access technologies can provide a joint solution.

## 2. Related Works

Contemporary digital media technology is formed based on digital information technology. Since the process of informatization of Western society started earlier, the digitization of media forms was earlier, Western scholars began to explore digital media technology from various fields and angles, and formed relatively comprehensive and in-depth theoretical research results [8]. From the macro perspective of digital media technology, many scholars have explored the impact of new digital media on the times and society. Marshall McLuhan proposed the concepts of “media as the message” and “media as human extension” in “Understanding Media—On Human Extension,” revealing the significance of media technology in changing human culture and life, and in the age of electronic media, the limits of time and space will be broken through, and the distance between people in time and space will shrink, thus forming a “global village” [9]. With its precise analysis and prediction, this work has become an enlightening theoretical foundation in the field of digital media. In “Virtual Art: From Illusion to Immersion,” I distinguish immersion in the digital media age from earlier forms of illusion art, draw on the actual works of contemporary artists and groups in the analysis, and summarize how to use 3D, IMAX, virtual reality, and other technologies. The means create immersive illusions and outline the impact of virtual reality on contemporary art perceptions [10]. In “Digital Survival,” it is described that in the world of the information age “information” is rapidly replacing material and becoming the basic exchange of society, and it explains that digital media technology based on the Internet and digital technology has an impact on human learning and work. In addition to the influence of entertainment and other lifestyle, it also has an important impact on the discussion and research on digital media technology [11].

The concept of the Internet of Things (IoT) was first proposed by Bill Gates, the president of Microsoft, but it was not given much attention at the strategic level because the development of the Internet was still in the rapid development stage and the related technology of IoT was not fully mature. Samie F took the transmission of radius sequence as the fundamental basis to express the distribution and heuristic algorithm of ant colony optimization, and then through the actual location of nodes [12]. The deployment is calculated in real-time to get the best node communication radius. This algorithm is heuristic, and although it can converge quickly to a more reasonable result, there is no way to ensure a continuous extension of the network lifetime because the nodes are the closest to the most correct solution and the algorithm is relatively difficult and costly. Many terminals, a small amount of data transmission, and low latency requirements are the characteristics of the current model of the Internet of Things. In terms of theoretical research, experts, and scholars such as Qiu et al. discussed the definition of IoT, technical directions, and the current development status of the IoT industry in China and worldwide and make a key analysis in each link of IoT industry chain and applications. The development status of the IoT application industry is also analyzed [13]. The Department of Technology and Economic Research of the Development Research Center of the State Council (2011) published a study on the development mechanism of China's IoT industry. The book presents the current situation of China's application of IoT technology and its problems in the sensor, cloud computing, smart grid, intelligent transportation, and electronic medical record industries [14]. The EPC field sees it as a mega-network covering everything in the world through next-generation information technology on the Internet. Any item in the network can interact with each other with data. In essence, it is the goal of information sharing by making objects automatically identify and transmit data through the Internet as a platform.

In summary, in terms of IoT technology, the promotion of the information age background has led to detailed and in-depth research both from a macro perspective and from a micro perspective of technology in the field of display, and on this solid foundation, there is still much room for improvement in research and practice regarding the use of technology in digital media content creation.

## 3. IoT-Based Digital Media Content Creation

### 3.1. IoT Model Design

The popularity and application of IoT technology have broken the traditional way of relying on a single information transfer between people. At the same time, the information transmission of IoT technology still needs to rely on information sensing equipment to achieve, with the help of equipment and processing [15]. The Internet of Things is essentially an extension and expansion of the Internet and communication networks. It mainly recognizes the physical world through intelligent devices and sensing technology and carries out knowledge mining, processing, and calculation based on network transmission and interconnection.

**Figure 1:**
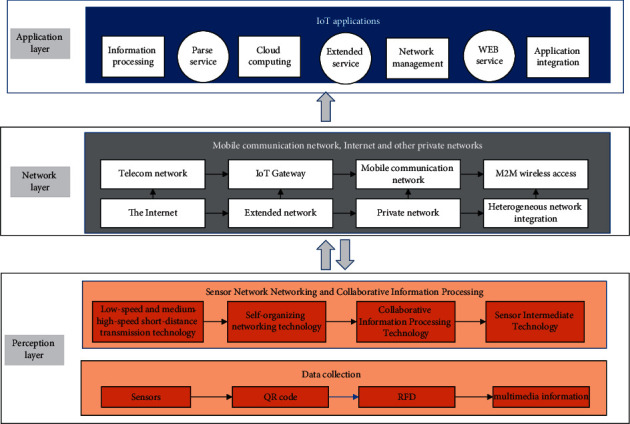
IoT network architecture diagram.

The roles and functions of each structure of IoT technology are also different. The perception layer is mainly used for information perception, such as information collection and identification; the network layer is mainly used for transmission of various information, while the application layer mainly presents the results of the final identification information in front of human beings to further promote the intelligent management of something by human beings, as shown in Figure 1.

As the basic unit of the neural network, the single neuron has self-learning and self-adaptive capability and has a simple structure and fast response time. The combination of single neuron and traditional PID controller to form a single neuron adaptive PID controller, to a certain extent, solves the traditional PID controller and is not easy to adjust parameters online in real-time; it is difficult to effectively control complex processes and time-varying system parameters and other problems. Neural networks are characterized by learning, and the basic means of learning is learning rules. The connection weights are changed utilizing learning so that the adaptive function of the single neuron controller is realized. The learning algorithm is the core of the whole controller and *w*_*i*_ is the rule that adjusts the connection weights and reflects its ability to learn. The goal of seamless linking and information exchange between them is to accurately manage and control the physical world in real-time based on scientific decision-making. The learning algorithm is as follows:

(1) $$\begin{array}{c} w_{i}\left(k\right)=w_{i}{\left(\kappa +1\right)}^{2}-\lambda \text{lin}\left(\kappa \right). \end{array}$$

**Figure 2:**
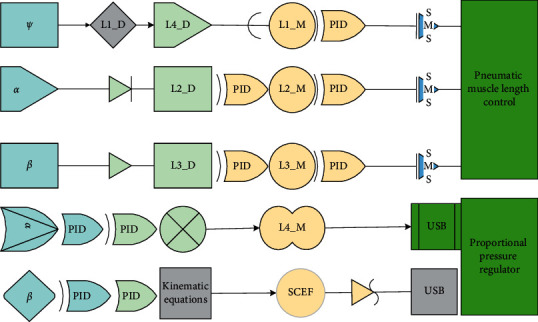
NB-IoT core network architecture diagram.

NB-IoT is an important branch of IoT technology, which has obvious differences from the traditional model in terms of voice service model and user distribution model. In traditional 2G, 3G, and 4G, the number of user accesses in base stations is controlled around 1000 and 400 in densely populated areas to ensure latency. However, NB-IoT does not require high latency, and it is dormant most of the time when there is no data interaction and activates the connection again when there is data exchange, which greatly improves the number of connections [16]. In terms of core network architecture, NB-IoT optimizes the EPC core network architecture, which can be known according to 3GPP's R13 protocol, and the principle of core network architecture is shown in Figure 2. Although there are certain differences in the way and focus of information transmission between the Internet and the Internet of Things, the ultimate purpose to be achieved is the same, which is to promote information transmission and communication between things and people. For the Internet of Things, this project the birth of technology has laid a solid foundation for the further improvement and development of Internet technology. The user-level management of network airports is performed in FDD mode, and the process is implemented by first accessing the NB RAN with airports and then accessing the C-SGN (Cellular IoT Service Gateway) network at the S1 interface. The core capabilities are shared to third-party platforms using standard API interfaces. The core capabilities mainly include contract data management and location update management (HSS), terminal SMS function interoperability (SMS-GMSC/IWMSC/SMS ROUTER), and service capability development platform (SCEF), etc.

Using the HOP-DV algorithm to introduce multiple mobile anchor points, since each WSN contagion has its self-assembled graph, they can use neighboring nodes to transmit data to the aggregation node, so the most economical way is to distribute a mobile access point per cluster; this access point acts as the aggregation node and mobile anchor point. The mobile anchor point can obtain its location arbitrarily, and after receiving the information from all nodes in the cluster, it feeds back the number of hops and the shortest path, moves to other locations in turn, and repeats the above communication process. The coverage of all nodes in the cluster and the nodes are limited by the communication distance, and the distance moved by the mobile anchor point cannot be too small at each time of generating the movement but must be evenly distributed within the cluster. NB-IoT does not require high latency. When there is no data interaction, it is in a dormant state most of the time, and the connection is activated again when there is data exchange. This design greatly increases the number of connections.

(2) $$\begin{array}{c} C=\frac{\sum_{i\neq j}\max\left(h_{ik}+h_{jk}\right)}{\sum_{i\neq j}\sqrt{\left(x_{i}+y_{i}\right)-{\left(x_{j}-y_{j}\right)}^{2}}}. \end{array}$$

In the formula, *h*_*ik*_ and *h*_*jk*_ represent the number of hops from any position *k* nodes to anchor points *i* and *j*, respectively, and the maximum value of the sum of the number of hops from all position nodes to 2 anchor points is obtained and taken as the reference value of the number of hops between 2 anchor positions. This solution solves the estimation method of the average hop distance C, but the accuracy is equivalent to the classical DV-HOP algorithm, because the positioning accuracy is low, and locating the unknown node position will produce a large error rate.

(3) $$\begin{array}{c} \Delta S\leq \gamma \frac{\Delta L}{kL}. \end{array}$$

**Figure 3:**
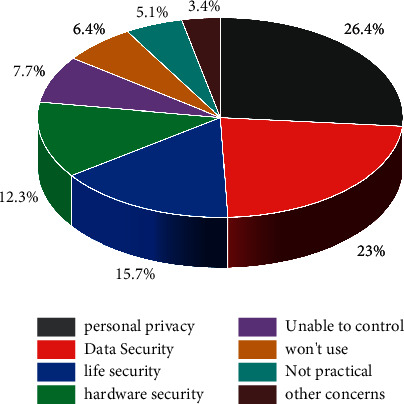
IoT security worry statistics results graph.

The cloud computing approach has many distinctive features with the development of the times and is widely used in various fields, with several main forms: processing, backup, multi-point storage, and large-scale centralized storage. This computing approach has a high value of use, can be used across platforms, and can be configured with servers, databases and core software, and other resources to meet the actual needs of applications. Based on the above advantages of cloud computing technology, cloud computing can provide stable and scalable basic services for storing and processing large-capacity WSN data. Contemporary society is the era of the Internet of Things; privacy issues are particularly important with the changing habits and learning paths of human life. From film, television, camera to video art, multimedia art, digital art, all are the progress of science and technology that inspired the birth of new art. The progress of science and technology has created new platforms and spaces for artists, which has greatly enriched the forms of artistic creation and promoted the development of art. In a questionnaire on “What should we worry about with the development of the Internet of Things,” personal privacy is the most important concern for contemporary people, followed by security. In addition to these two reasons, the following issues are operating system, application vulnerability, unauthorized access, identity impersonation, etc. The detailed statistics are shown in Figure 3. For WSN with IoT-NB technology, the sensing nodes have a huge amount, high data redundancy, simplicity, weak computing power, security issues, and drawbacks in the case of relying only on cloud computing has a huge impact and threat.

### 3.2. Digital Media Content Creation Model Construction

Digital Media is not simply a superimposition of digital technology and traditional media, but full integration of digital media and communication content. Digital media is more commonly referred to as “New Media” in the discourse of Western researchers. The difference between “new media” and “old media” is that, on the one hand, the plural form of the word “media” means that “new media” is no longer seen as a single media organization or media structure (e.g., print media, radio, and television media, etc.), but as a collective concept that divides all forms of digital media into one [17]. On the other hand, the “new media” integrates the content and characteristics of all existing media forms and adds digital, interactive, two-way communication characteristics to them. The application of this new form of digital media and novel technology in art has a broad development prospect and market appeal. It is widely used in various exhibition halls, and digital media art is used in digital display, 3D virtual display, interactive display, and wayfinding system design. Digital media can be seen as a system that covers digital media organizations, products, technologies, contents, networks, and terminals and is led by government departments, enterprises, and individuals, with the means of computer technology and network information technology; digital media art contents are disseminated through the Internet, mobile communication networks and digital TV networks, on computers, mobile personal terminals, wearable devices, etc. On the digital terminal, it presents various websites, multimedia, computer animation, electronic games, interactive novels, interactive images, interactive devices, and virtual reality products.

**Figure 4:**
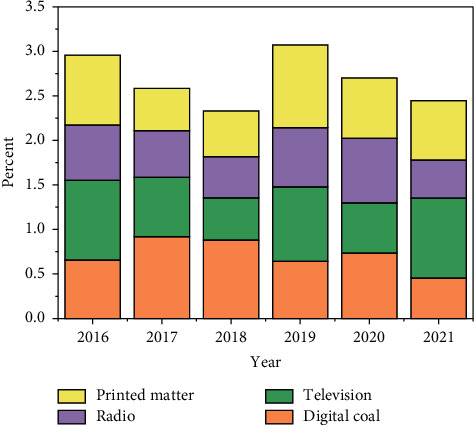
2016–2021 average daily time and timeshare of people using each mainstream media.

As information technology continues to evolve in the digital age, traditional media have subsequently taken on their digital new forms. Web-based communication methods are becoming increasingly popular, personal digital terminals are gradually entering people's lives, and the distance between digital media and human beings is gradually being brought closer. As Negroponte said in “Digital Survival,” “the development of information technology will bring revolutionary changes to our way of life.” Information will no longer be “sold” to “consumers,” but humans will carry information provided by “digital handymen” and participate in the interaction process, and the mass media will gradually evolve into a two-way communication media with personal characteristics [18]. The mass media has gradually evolved into a two-way communication media with personalized characteristics. Chinese users have gradually shifted from traditional media to digital media and are spending more time on digital media than ever before. According to a statistic by Ariadne, from 2016 to 2021, the average daily use of digital media per capita in China increased from 1.78 hours to 3.08 hours, and the proportion increased from 35.8% to 50.4%, while the average daily exposure to traditional media per capita did not change significantly, as shown in Figure 4.

oT-NB can be called a simplified LTE network. It is characterized by wide coverage and large connections. It plays a very important role in the analysis and evaluation of IoT-NB IoT coverage and the planning of network capacity and scale effect. In the past, the exhibits on display were mainly in the form of physical objects. Due to their preciousness and state of conservation, these objects are not allowed to be touched by the public at close range, and there are restrictions on the time and place of their display. When the public goes to museums, they often encounter gaps in the content of the exhibits due to their condition or loans to other museums, which greatly affects visitors' interest and their desire to explore further in the exhibition activities [19]. With the addition of digital media technology, the exhibits start to develop from materialization to dematerialization and are presented in digital form in front of the visitors. Not only are the digital exhibits not easily damaged, is the display environment not restricted, and can the audience interact with them at zero distance, but also the same content can be displayed in multiple places at the same time, forming the sharing of resources. In addition, the digital exhibits will be a dynamic interpretation of static physical exhibits, making the exhibit content more vivid and comprehensive, stimulating the interest of the audience, thus improving the dissemination efficiency of museum display content.

A direct display scheme to solve the equations would make the algorithm less efficient, and therefore an acceleration of the algorithm is needed. The additive splitting algorithm (AOS) algorithm is a semi-implicit scheme for solving the nonlinear diffusion equation, where the image data of the kth + 1st iteration is not directly generated from the data of the kth iteration but is given indirectly through a matrix-vector equation. Since the stability of this semi-implicit scheme is not affected by the iteration step, a larger time step can be set to obtain a faster update speed. The rows and columns of the image are first diffused in one dimension using the semi-implicit scheme.

(4) $$\begin{array}{c} I_{\kappa +1}=\sum_{d=1}^{2}\left(A_{d}+2\tau I_{\kappa }\right)\cdot I_{\kappa +1},\\ C_{l}=\frac{2L_{\min}}{L_{\max}-L_{\min}}. \end{array}$$

**Figure 5:**
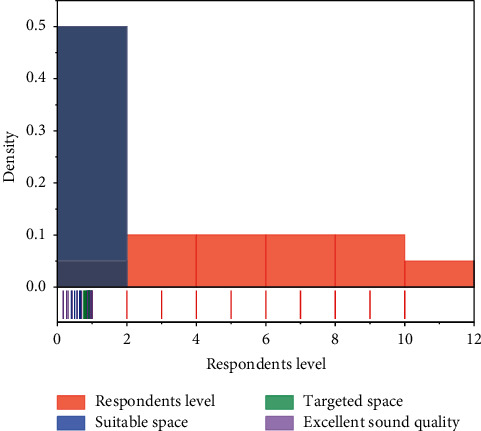
Respondents' level of need for a sense of presence in the space itself.

With the continuous progress of technology, people's information concept is also constantly updated. People hope to meet the needs of information acquisition through various dynamic image information display channels and have a variety of sensory experience. This is something that cannot be given by traditional exhibition activities. In the era of digital media technology, the popularity and development of digital media technology have overturned the form of media and formed a unique form of information dissemination, and the exhibition activities have also changed significantly with the intervention of digital media technology. The purpose of immersive experience is to make the audience be completely submerged in the virtual scene displayed by digital media technology [20]. It is revealed that media technology is of great significance to the change of human culture and life, and it has made an in-depth interpretation of media technology in various eras. It is believed that in the era of electronic media the time and space limitations will be broken, and the space and time distance between people will continue to shrink and form a “global village.” If some parts of the space itself distract the viewer's attention, it means that the viewer is not fully engaged in the virtual scene during the visit, which results in a weakened sense of psychological immersion. In the evaluation of the importance of each element, it can be found that the elements that highlight the image of the space itself are no longer considered important to enhance the experience of digital display, while the physical environment with low presence has become a new demand for visitors, as shown in the Figure 5.

Therefore, the principle of virtualization guides architects to weaken people's sense of place in physical space when designing space. On the one hand, they can achieve indirect blurring of material space at the visual level using special interface materials, environmental illumination, or environmental color treatment, so that people cannot judge the boundaries and environment of space in space, nor can they clarify orientation and direction. On the other hand, digital photoelectric technology can be used to create virtual boundaries, which can directly realize the materiality of some elements in the physical space from a technical perspective.

## 4. Analysis of Results

### 4.1. IoT Model Results

Wireless sensor network technology is a distributed sensor network technology that senses and checks external world sensors. Its communication is achieved mainly by wireless means, with a very flexible network setup that can be changed at will, as well as by using wireless or wired connections to the Internet. Wireless connectivity is a multi-hop organizational network model that combines tens of thousands of sensing nodes freely to form a network. The technology is characterized by its large scale, self-organization, and dynamic form, making it useful for a wide range of applications, such as medical, military, and environmental monitoring. However, the traditional wireless sensor network node deployment method has problems such as low coverage and long and unstable mobile steps. This study proposes a wireless sensor network node redeployment method based on frog hopping algorithm, simulating the way of frog flock foraging, classifying the subpopulation of network nodes, realizing the whole process of information transfer combined with global information exchange, mixing all the frogs within the subpopulation, then classifying the subpopulation, collecting a certain place in the network coverage, using the nearest node to collect relevant data information, reducing the repetitive collection situation occurs, calculating the deployment of wireless network nodes, within the scope of the network area, divided into small areas of equal size, where the nodes can cover the area, that is, the area being monitored, the need for special monitoring, to complete the wireless sensor network node redeployment. This algorithm is heuristic, although it can quickly converge to a more reasonable result, but because the node is the closest to the most correct solution, and the algorithm is relatively difficult and the cost is relatively high, there is no way to ensure a continuous extension of the network service life.

The coverage degree is an important parameter to measure the node deployment of the sensing network, i.e., the area covered by all nodes and the total ratio of the target area. Therefore, node redeployment starts with the calculation of the coverage of the optimal solution node, and the node redeployment is achieved by searching the coverage area through the coverage rate. If a certain area is to be focused on monitoring within the detected area, the calculation method at this time is to be calculated using different weights. If only one of these areas is needed for special monitoring, this node coverage area is expressed by the following formula:

(5) $$\begin{array}{c} S_{\text{all}}=S_{\text{area}}-\left(w+1\right)\times S_{i}. \end{array}$$

**Figure 6:**
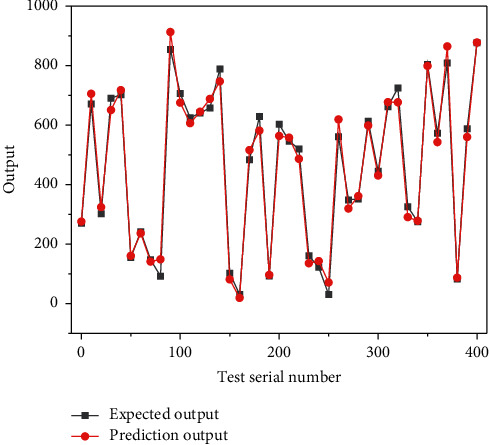
Schematic diagram of predicted and actual output distribution.

The above equation represents the area of the key monitoring area and represents the weight, which can be changed according to different specialty areas. The random forest (RF) algorithm, first proposed by Tin Kam in 1995 and studied in more depth by Breiman, enables pattern classification and regression prediction of the data to be input. RF successfully implements a combination of bootstrap strategies and random subspace methods. Since the decision tree is built very fast, the training of the decision tree is much faster than training an artificial neural network. To implement the random forest-based greenhouse temperature prediction, three environmental factors, namely, air temperature, air humidity, and light intensity in the greenhouse, are selected as model inputs to build the random forest prediction model. In this paper, the number of random forest decision trees is 500, which helps to enhance the generalization ability of the model calculation process. The roles and functions of each structure of the Internet of Things technology are also different. The perception layer is mainly used for information perception, such as information collection, identification, etc.; the network layer is mainly used for the transmission of various information, and the application layer is mainly used for information. The 2000 sets of data are selected as the training set and 400 sets as the prediction set. As shown in Figure 6, the comparison between the predicted output and the actual output shows that the predicted output is very close to the actual output, and the calculated RMSE is 0.0183, which shows that the prediction accuracy of the random forest model is good from the intuitive point of view.

From the above experimental results, the random forest prediction model has a low error in the prediction experiments of greenhouse temperature, which is a key factor for tomato growth, and its root mean square error of prediction is about 0.0183, which is a low prediction error and a more accurate prediction of greenhouse temperature trends. Coverage is a measure of test integrity, reflecting the energy consumed by the network coverage area, and the degree of node redundancy. The higher the coverage is, the less redundant the network nodes are, while the opposite is true for the redundancy of the network nodes.

**Figure 7:**
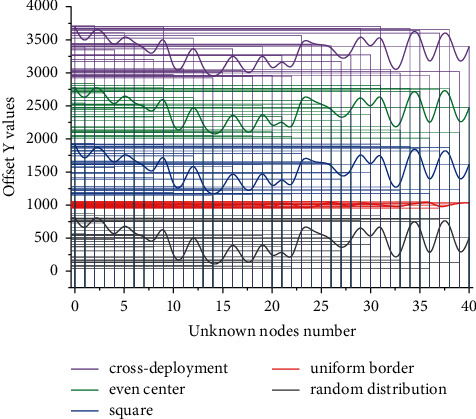
Comparison of the effect of different anchor deployment methods on the error rate.

During the process of recording the single-hop or multi-hop information sent at each position where all nodes stay, the minimum number of node hops is sent, that is, the shortest path. And the initial node communication radius *R* is 6 m. The simulation results show that the error rate is lower compared with the HOP-DV (random deployment) and the improved DVHOP-NBIOT (uniform deployment at the boundary) under the condition that the number of unknowns (12) is equal, the communication radius (6) is fixed, and the number of unknown nodes (31–13) is unequal. The accuracy of the unknown node localization is greatly improved. A comparison of the effect of different mobile anchor deployment methods on the error rate is shown in Figure 7.

The deployment method of mobile anchor locations has a direct impact on the localization accuracy of unknown nodes. The DVHOP-NBIOT optimized localization algorithm, from the perspective of the localization error, produces lower results using several deployment methods such as center uniform, square, and cross than the boundary average deployment method. The center-uniform deployment method gives the best results with an average error rate of 18.66%. In the HOP-DV classical algorithm, irregular location deployment always occurs, which leads to unstable and large differences in the localization accuracy between the anchor nodes to be located and the unknown nodes. The result of the final identification information is presented to humans, which further promotes the intelligent management of something by humans. In summary, the DVHOP-NBIOT optimization algorithm makes the best use of the self-localization capability of NB-IoT, the background computing capability, and further combines the deployment optimization of the original location of anchor points to calculate the unknown node positioning, and a comparative analysis based on the original DV-HOP method finds that the error rate is reduced to a great extent.

## 5. Digital Media Content Creation Simulation Experiment

The digital display is based on the theoretical knowledge of display design and digital theory, based on modern information technology and other related fields (such as museums and exhibitions, etc.) to achieve the purpose of display for the public. Nowadays, digital image technology is maturing and has been popularized in China. Contemporary exhibition halls are also gradually adopting this new display technology, changing from static display to dynamic display, and a certain part of the halls even takes digital image technology to the extreme, developing into a multi-sensory interactive display mode. The growing maturity of digital technology has brought a revolution to the exhibition industry and brought a deeper impression and more vivid experience to the public. Digital display design will open a new chapter for this modern exhibition industry.

Traditional display activities are mainly in the form of static physical display, and browsing panels and exhibits are the main activity of the audience, so visual is the main way for people to get information. On the one hand, the visual experience alone will affect people's sense of visiting experience and the degree of memory of information. Through the experiment, it was found that 94% of the information received came from visual, 11% from auditory, and 6% from the smell, touch, and taste perception, which indicates that visual and auditory are still the two main ways for people to obtain information, but the role of other senses also assists people in receiving information. In addition, the study also found that people's memory of information through reading accounted for 10% of the total, the memory of hearing for 20%, the memory of visual for 30%, the memory of listening while watching for 50%, the memory of telling by oneself for 70%, and memory of combining and acting on various ways for 90%, which indicates that the more perceptions involved in information acquisition, the stronger people's memory of information will be.

**Figure 8:**
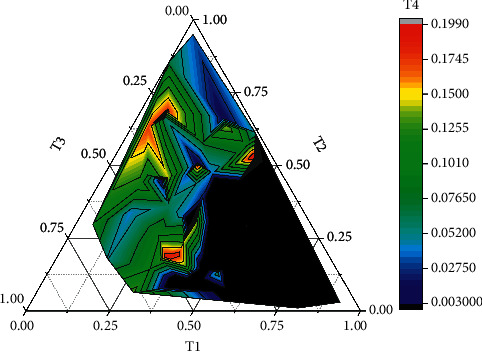
System performance test results.

This section firstly introduces the basic concept of a variational method for image processing, the variational principle, and Euler's equation in detail and investigates the principle of a variational method for image restoration by introducing the variational problem in image restoration; i.e., the problem is transformed into a generalized function on the extremum problem to find the minimum value of the problem. The results show that the TV model has the strongest denoising ability, the P-M model has the strongest edge-holding ability, and the linear model is in the middle of the range, with a slightly weaker denoising ability than the TV model and weaker edge-holding than the P-M model. Finally, Chen's coupled PDE model is studied, which lays the foundation for multi-physics field coupling denoising. The system performance results are obtained by testing three sets of experimental data, as shown in Figure 8.

**Figure 9:**
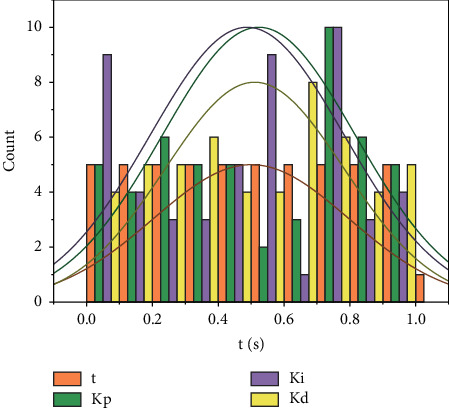
System parameter self-tuning curve.

According to the consumption ratio, the consumption of lookup tables and flip-flops in the project is 38.02% and 14.89% respectively, both below 70%. The clock management MMCM and phase-locked loop PLL use 3 and 1, respectively, and there are still scalable parts of the clock. The global buffer BUFG uses 10 units, and the remaining 22 units can be used to reduce the signal transmission delay and increase the signal transmission frequency. In summary, the FPGA chip is fully capable of implementing this system project. Cloud computing methods mainly include processing, backup, multi-point storage, and large-scale centralized storage. This computing method has high use value, can be used across platforms, and can be configured with servers, databases, core software, and other resources to meet the actual needs of applications. As shown in Figure 9, due to the large error at the beginning, the single-neuron network adjusts the three PID parameters online quickly with a large amount of adjustment, so that it can compensate for the temperature error quickly and track the input signal as soon as possible; when the error becomes smaller, the single neuron adjusts the three PID parameters with a small amount, so that it can track the input signal stably and maintain the stability of the control.

This section presents the systematic implementation of the algorithm by modularizing the system and summarizing the design structure and design methodology of each module in the system in each subsection. Simulation of several important modules, such as data storage and data processing modules, is also carried out and compared with the software implementation to highlight the advantages of the hardware implementation of the algorithm. Finally, the system is implemented on the board to complete the whole algorithm from design to implementation, which proves that the algorithm hardware implementation has the algorithm enhanced image function and meets the algorithm real-time requirements.

## 6. Conclusion

This paper designs a digital media content creation method based on NB-IoT IoT technology by studying the current stage of IoT technology, new energy technology, database technology, Web technology, and fuzzy control technology in the digital media field. The system abandons the traditional wireless communication technologies such as ZigBee and Wi-Fi, which are widely used, and chooses NB-IoT narrow-bandwidth IoT technology as the communication module of the whole system, collects the sensors of each environmental factor at the terminal, sends the collected data to the remote server through the NB-IoT network, stores them in the database, and realizes the web page of B/S structure with Java technology. The administrator can log in to the web page through a PC or mobile terminal to realize intelligent management. In terms of control, the design adopts a fuzzy control algorithm to realize the regulation of external environmental factors and establishes the ARIMA model to predict the future environmental change trend and provide data support for intelligent management. There are still many shortcomings in the theoretical and practical research results in this paper. The topic of how to promote the development of the new media industry by promoting the development of digital media advertising is a grand and long-term topic that needs to be studied continuously. In the future research process, the author will conduct richer and more in-depth theoretical research to supplement and sublimate the research of this paper.

## Data Availability

The data used to support the findings of this study are available from the author upon request.
